# IMPLEMENTING SMART AMBIENT BRIGHT LIGHT (SABL) IN NURSING HOME RESIDENTS WITH DEMENTIA: CHALLENGES AND STRATEGIES

**DOI:** 10.1093/geroni/igad104.2292

**Published:** 2023-12-21

**Authors:** Ying-Ling Jao, Julian Wang, Yo-Jen Liao, Diane Berish, Marie Boltz, Margaret Calkins

**Affiliations:** Pennsylvania State University, State College, Pennsylvania, United States; Pennsylvania State University, State College, Pennsylvania, United States; Pennsylvania State University, State College, Pennsylvania, United States; Pennsylvania State University, State College, Pennsylvania, United States; Pennsylvania State University, State College, Pennsylvania, United States; IDEAS Institute, Cleveland Heights, Ohio, United States

## Abstract

Effective non-pharmacological interventions are critically needed to reduce agitation for nursing home residents with dementia. Lighting interventions (LIs) improve agitation among persons with dementia. However, LIs have not been widely implemented in “real-world” care settings, and a feasible implementation approach to ambient LIs has not been established. This study describes challenges and strategies in implementing the smart ambient bright light (SABL) to provide auto-controlled, consistent indoor lighting that incorporates natural daylight. This SABL includes tunable LED lights, photosensors, and controllers and has a pre-programmed 24-hour control schedule for illuminance. The SABL will be installed in participants’ bedrooms, dining areas, and activity rooms for four weeks. Key challenges include nursing home leadership team’s concerns about compliance with regulations and increased staff workload. Our implementation strategies include 1) engaging the nursing home stakeholders early on to address their concerns, 2) setting up an advisory board at each facility to involve the administrator, director of maintenance, and frontline healthcare workers to ensure the implementation protocol is congruent with their regulations, policy, and staffing, 3) tailoring lighting schedule based on the care routine and resident schedules, and 4) monitoring the SABL operation on-site and remotely. Moreover, the study incorporates a mixed methods design to interview staff/administrators and evaluate the intervention’s acceptability, feasibility, and appropriateness from their perspectives. This is the first study that addresses the implementation strategies of LIs. Findings will establish evidence-based implementation strategies and the best design for SABL to reduce agitation for nursing home residents with dementia.

